# Extraction, content determination, component analysis, and pharmacological action of Hedysari flavonoids: a review of research progress

**DOI:** 10.3389/fphar.2025.1517832

**Published:** 2025-04-10

**Authors:** Cancan Huang, Haiyan Mao, Wenwen Wan, Chunzhen Ren, Xiujia Ji, Bin Yue, Xiaohua Zhang, Quansheng Wu

**Affiliations:** ^1^ Clinical School of Traditional Chinese Medicine, Gansu University of Chinese Medicine, Lanzhou, China; ^2^ Department of Traditional Chinese Medicine, Gansu Provincial People’s Hospital, Lanzhou, China; ^3^ School of Traditional Chinese and Western Medicine, Gansu University of Chinese Medicine, Lanzhou, China

**Keywords:** Hedysari flavonoids, content determination, structural analysis, anti-osteoporosis, anti-oxidation, improvement in fibrosis

## Abstract

Radix Hedysari is a traditional Chinese herbal medicine and food. It has a long history of clinical application and is used to improve health and treat various diseases. Hedysari polysaccharides, flavonoids, saponins, and alkaloids are the main components of Radix Hedysari. Hedysari flavonoids are the most important natural active ingredients in Radix Hedysari and have a variety of pharmacological effects. At present, Hedysari flavonoids have shown great application prospects in the development of drugs for the treatment of pulmonary fibrosis, osteoporosis, skeletal muscle injury, atherosclerosis, hepatic fibrosis, immune regulation, hypoglycemia, and renal fibrosis. This paper reviewed the extraction, separation, and content determination methods and chemical constituents of flavonoids from Radix Hedysari. The core electronic databases used for literature retrieval included Baidu Literature, Baidu Literature, WangFang Datas, CNKI, VIP Datas, PubMed, Google Scholar, and Web of Science. “*Radix Hedysari*”, “Hong qi”, “Flavonoid”, “*Hedysari* Flavonoids” and “pharmacological effects”, “extraction” and “structure” were used as the search terms from database creation to 30 October 2024. Multiple studies have shown that Radix Hedysari flavonoids have important pharmacological effects, such as cytotoxic effects on tumor cells, by inhibiting tumor cell growth, inducing tumor cell apoptosis, and enhancing immune function. The antioxidant effect, through the regulation of LDH, SOD, malondialdehyde and other enzymes and the expression of antioxidant-related factors, can improve osteoporosis by promoting osteoblast proliferation and differentiation, reducing calcium loss, increasing bone mineral density, maintaining bone balance and improving bone quality. These effects include reducing oxidation; preventing thrombosis; enhancing endothelial function; regulating blood lipid levels to improve anti-atherosclerosis, anti-pulmonary fibrosis and liver fibrosis performance; improving atherosclerosis; reducing skeletal muscle damage; and exerting immunomodulatory effects, such as regulating various cytokines, immune cells, immune organs and related signaling pathways. This work provides a theoretical foundation for further studies on the structure, mechanism and clinical application of Radix Hedysari flavonoids.

## 1 Introduction

Hedysarum polybotrys Hand.-Mazz. (Fabaceae) is the source of the botanicial drug Hedysaria radix (root) used as a traditional Chinese herbal medicine and food. Noted for its sweet taste, it has various medicinal effects: invigorating qi, promoting yang, strengthening the surface, stopping sweating, promoting diuresis, detumescence, fluid nourishment, blood promotion, stagnation relief, arthralgia dredging, poison support, and pus expulsion (Feng et al., 2021). Flavonoids have been identified as crucial compounds formed by linking two phenolic hydroxyl benzene rings (A and B rings) via the central three carbon atoms, which are vital for the biological activities of plants. Xu et al. (2018) used high-performance liquid chromatography (UPLC) to analyze the fingerprints of 1-year-old and 2-year-old Radix Hedysari. The results revealed 6 chromatographic peaks of 1-year-old and 2-year-old Radix Hedysari, which were attributed to calycosin-7-O-β-D-glucopy ranoside, formononetin, soyasaponin I, formononetin, isoastragaloside II, and astragaloside I, respectively. Huang et al. (2019) used a rapid and universal method involving a cysteine-rich peptide (CRP) fingerprint, which can accurately and quickly detect the chemical constituents of the natural drug Radix Hedysari, mainly polysaccharides, flavonoids, and saponins. Pharmacological studies demonstrate antioxidant, immune regulation, hypoglycemic, and other effects. While current research has explored mainly Radix Hedysari flavonoid components, Radix Hedysari flavonoids exhibit potent pharmacological effects, including cytotoxic effects on tumor cells, anti-oxidation, pulmonary fibrosis improvement, anti-osteoporosis effects, and skeletal muscle injury reduction. In recent years, some studies have compared Hedysari flavonoids with Astragalus flavonoids and reported that Hedysari flavonoids are more effective than Astragalus flavonoids in cytotoxic effects on tumor cells and anti-oxidation activities. Therefore, the use of Hedysari flavonoids has attracted increasing attention from scholars at home and abroad. In summary, this paper reviews the flavonoid extraction, separation, and pharmacological effects of Radix Hedysari. It also analyzes and summarizes the potential value of Radix Hedysari flavonoids, providing insights for their application in the food, healthcare, and pharmaceutical fields.

**FIGURE 1:**
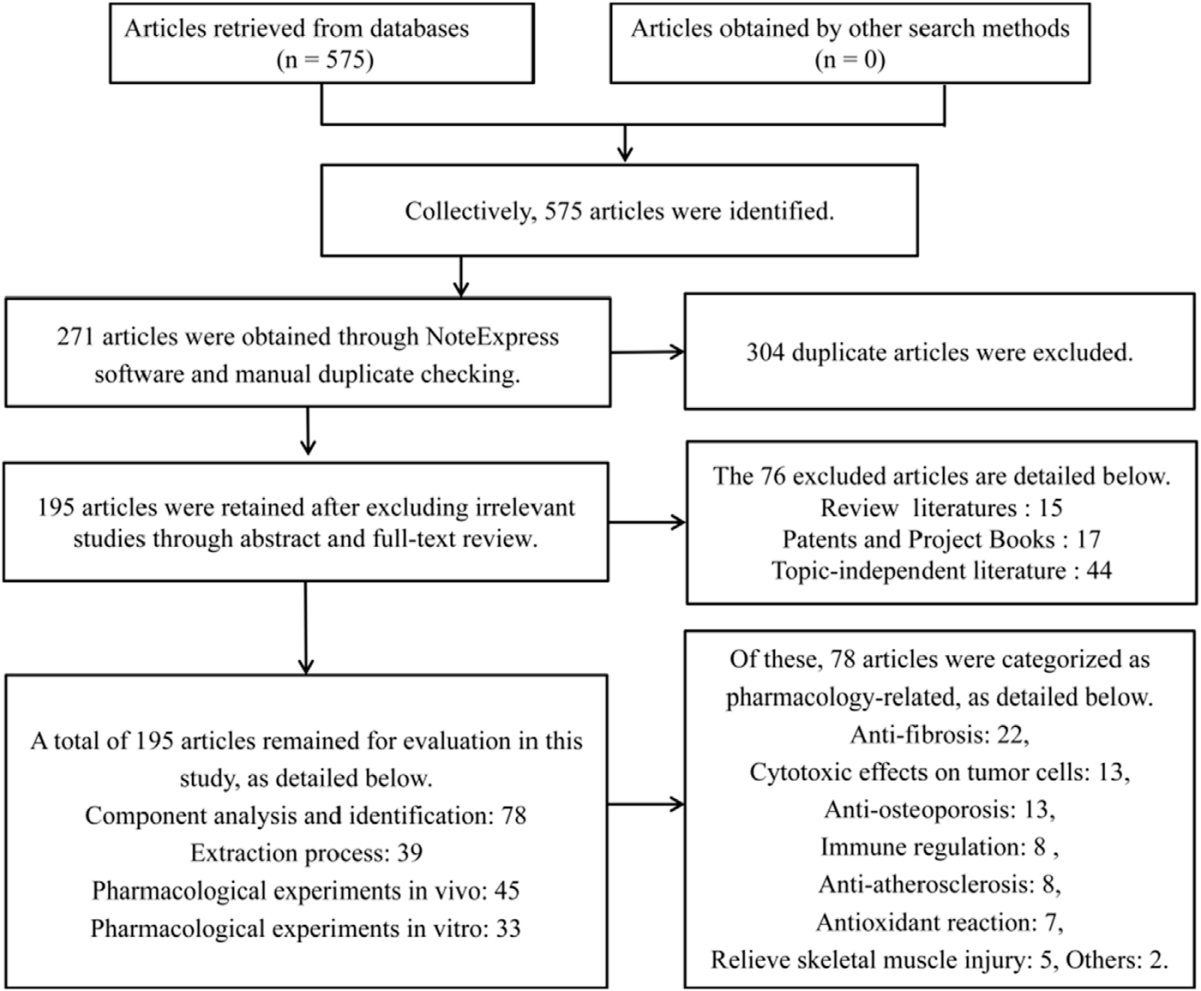
Literature retrieval and screening process.

This manuscript was retrieved in the form of a database search. The search terms are in the form of subject words combined with free words. We systematically searched Baidu Literature, WangFang Datas, CNKI, VIP Datas, PubMed, Google Scholar, and Web of Science. Using “*Radix Hedysari*”, “Hong qi”, “Flavonoid”, “*Hedysari* Flavonoids” and “pharmacological effects”, “extraction” and “structure” as the search terms from database creation to 30 October 2024. Collectively, 575 articles were identified. After removing duplicates 271 and irrelevant articles 76, a total of 195 articles remained for evaluation in this study, as shown in Figure 1. We included *in vitro* studies and animal model studies. We extracted study details, including relevant information on the pharmacological action and chemical attributes of Hedysari flavonoids, as well as the study status.

## 2 Extraction and separation of flavonoids from Radix Hedysari

Flavonoids hold significant medicinal value, with natural drugs serving as crucial sources. Presently, various methods are employed for extracting flavonoids from traditional Chinese medicine, including solvent extraction (Huang, 2019), ultrasonic extraction (Yusoff et al., 2022), microwave-assisted extraction (Gu et al., 2020), alkali dissolution and acid precipitation (Chen et al., 2020b), enzyme-assisted extraction (Lan and Chen, 2017), and supercritical fluid extraction (Memon et al., 2016), as well as emerging techniques such as subcritical water extraction (2021) (SWE), Tween 60 synergistic microwave extraction (Jiang et al., 2020), the decompression internal boiling method (Wang, 2019), and flash extraction technology (Chen et al., 2020a). Appropriate extraction and separation techniques are vital for swiftly obtaining target components. Despite advancements, the extraction and separation of flavonoids from Radix Hedysari remain challenging, with four primary extraction methods being utilized, as shown in Table 1.

**TABLE 1:** Different extraction methods for Radix Hedysari flavonoids.

Extraction method	Solvent	Advantage	Disadvantage	References
Ethyl acetate Soxhlet extraction method	Ethyl acetate Soxhlet	The solvent utilization rate is high and the extraction efficiency is good	The extraction time is long, and the equipment requirements are high	Li et al. (2009)
Ultrasonic extraction method	alcohol	The equipment is easy to operate, the extraction is rapid and efficient, the time and energy are saved, and the extraction effect is good	The cost of ultrasonic equipment is high, the stability of components is relatively low, and it generates noise pollution	Hailixi et al. (2009), Yuan et al. (2019a), Kou et al. (2020)
Reflux extraction method	alcohol	Simple, high extraction efficiency	Longer extraction time and high solvent consumption	Zheng et al. (2011)
Enzymatic extraction method	——	Exextraction was efficient, mild conditions, environmentally friendly and good selectivity	High cost, poor enzyme stability, specificity restriction, complex enzyme preparation and purification, and difficult recovery and reuse	Chen (2015)

### 2.1 Ethyl acetate soxhlet extraction method

In the initial phase, Li et al. (2019b) employed ethanol reflux, alkali-soluble acid precipitation, and ethyl acetate Soxhlet extraction for flavonoid extraction from Radix Hedysari. The ethyl acetate Soxhlet extraction method exhibited favorable extraction efficiency among these methods. Specifically, a mixture of ethyl acetate, chloroform, and water (4:9:1) served as the expansion system for identifying flavonoids in Radix Hedysari. This method is color-sensitive, facilitating total flavonoid identification in Radix Hedysari.

### 2.2 Ultrasonic extraction method

The ultrasonic extraction method stands out for its simplicity in terms of equipment, ease of operation, swift extraction process, high yield, and absence of heating, thereby safeguarding fragile components and conserving time and energy while ensuring a high extraction rate. It operates by using mechanical, cavitation, and thermal effects to swiftly release, diffuse, and dissolve cell constituents. In previous studies, Hailixi et al. (2009) achieved 0.07418% total flavonoid extraction from 70% ethanol via ultrasonic ethanol extraction. Kou et al. (2020) employed ethanol ultrasonic extraction followed by polyester amide powder adsorption chromatography and methanol elution to determine the total flavonoid content in Radix Hedysari from diverse regions. They reported that there were some differences in the contents of total flavonoids, total saponins, total polysaccharides, and 9 beneficial inorganic elements in different areas of Radix Hedysari. Yuan et al. (2019a) conducted ethanol ultrasonic extraction of total flavonoids from Radix Hedysari and assessed their antioxidant activity via the diphenylpicrylhydrazyl (DPPH) method. These results indicated that the solution of total flavonoids from Radix Hedysari obtained via ethanol ultrasonic extraction exhibited robust antioxidant capacity *in vitro* within a concentration range of 1–10 mg/mL, indicating a discernible dose-response relationship. Widely embraced in medicinal material flavonoid extraction, ultrasonic extraction effectively preserves flavonoid biological activity, mitigating incomplete extraction or active ingredient degradation.

### 2.3 Reflux extraction method

Zheng et al. (2011) conducted reflux extraction of Radix Hedysari using 50% ethanol. The chemical constituents were isolated through repeated silica gel, gel, reversed-phase column chromatography, and preparative liquid chromatography, with compound structures identified via spectroscopic methods. The extracted compounds included isoflavones such as formononetin, formononetin-7-O-glucoside, calycosin, and calycosin-7-O-glucoside; flavonoids such as 7,4′-dihydroxyflavone; dihydroflavonoids such as 7,4′-dihydroxy flavonoids; and phenylpropanoids such as n-hexadecyl ferulate and ferulic acid. While this method is simple and efficient, enabling effective flavonoid extraction from Radix Hedysari, it does entail prolonged extraction times and significant solvent consumption.

### 2.4 Enzymatic extraction method

Chen et al. (2015) employed a quadratic general rotary combination design to optimize the enzymatic extraction process of Radix Hedysari. For the first time, they established a mathematical model for ultrafiltration purification of fibrous rhizome medicinal materials, represented by Radix Hedysari. Additionally, they developed a BP neural network prediction model with the transfer rates of total polysaccharides, total saponins, and formononetin as output variables. This model exhibited high prediction accuracy and demonstrated effective prediction capabilities.

## 3 Content determination of flavonoids in Radix Hedysari

### 3.1 Determination by high-performance liquid chromatography (HPLC)

Currently, the determination of flavonoids in Radix Hedysari medicinal materials, processed products, and Radix Hedysari preparations relies primarily on HPLC. This method is valued for its simplicity, reliability, accuracy, excellent repeatability, and stability. It is the most commonly employed and widespread content determination method.

#### 3.1.1 Radix Hedysari medicinal materials

Li et al. (2019b) employed HPLC to assess calycosin and formononetin levels in Radix Hedysari, confirming the suitability of HPLC for evaluating key components. Ye et al. (2018) identified four isoflavones (calycosin-7-O-β-D-glucopyranoside, ononin, calycosin, and formononetin) in 1-year-old and 2-year-old Radix Hedysari from different habitats in Gansu Province via an HPLC method. A similar quality was observed in 1-year-old and 2-year-old Radix Hedysari on the basis of the analysis of the contents of the four isoflavones. Yang et al. (2017) proposed a quantitative method of multi-component single-marker (QAMS) analysis for the four flavonoids formononetin, calycosin, genistein, and formononetin in Gansu Radix Hedysari using HPLC. The application of QAMS demonstrated the potential for more comprehensive quality control of Radix Hedysari, providing a scientific basis for its thorough development and utilization. Li et al. (2017a) employed HPLC to determine the contents of four isoflavone components (calycosin, calycosin-7-O-β-D-glucopyranoside, formononetin, and formononetin) in Radix Hedysari samples from three producing areas (Wudu, Dangchang, and Longxi) across different months (July, August, September, and October). The characteristic component of Radix Hedysari samples from Wudu, Dangchang, and Longxi (July, August, September, and October) was found to be calycosin-7-O-β-D-glucopyranoside. Liu et al. (2016) initially developed an HPLC-DAD method for determining adenosine, isoliquiritigenin, medipigenin, and γ-aminobutyric acid (GABA) in Radix Hedysari. A relatively high content of the flavonoid medipigenin was identified, providing a novel perspective for elucidating the pharmacodynamic material basis of Radix Hedysari.

#### 3.1.2 Processed products of Radix Hedysari

Li et al. (2017b) utilized HPLC to analyze the calycosin and formononetin contents in microwave-processed Radix Hedysari products and reported values of 3.0984 μg g^–1^ calycosin and 42.314 μg g^–1^ formononetin. These findings are in accordance with the 2015 edition of the Pharmacopoeia of the People’s Republic of China, confirming the simplicity, feasibility, and reproducibility of the method. Different processing techniques also have varying effects on the flavonoid content of Radix Hedysari. Bai et al. (2019) employed HPLC to assess the total flavonoid and formononetin contents in honey-fried Radix Hedysari, integrating original and processed samples. The results revealed significant enhancements in total and index component levels compared with those of traditional honey-fried Radix Hedysari decoction pieces, suggesting the viability of this modern processing technology. Similarly, using HPLC, Niu et al. (2017) compared honey-fried Radix Hedysari with traditional stir-frying, baking, and microwave processing. Their analysis revealed the highest calycosin and formononetin contents in roasted Radix Hedysari, offering an experimental foundation for quality control, standardized processing of processed Radix Hedysari products, and clinical application insights.

#### 3.1.3 Radix Hedysari preparation

Radix Hedysari, which serves as both a medicinal and edible material, is extensively applied in food and healthcare products and is commonly employed as a primary component in Chinese patent medicines. Zhao (2014) utilized HPLC to assess the levels of calycosin-7-O-β-D-glucoside, genistein, formononetin, and pterocarpin simultaneously in both Radix Hedysari medicinal materials and Radix Hedysari oral liquid, thereby establishing a theoretical framework for the quality control of these substances.

### 3.2 Ultraviolet spectrophotometry

Ultraviolet spectrophotometry is a commonly employed method for determining total flavonoids in medicinal materials, and it is recognized for its ease of use, dependable results, and cost-effectiveness. Cheng et al. (2018) utilized this technique to measure the total flavonoid content in Radix Hedysari polybotrys sleep-promoting granules, yielding a value of 6.456 mg/g. Ye et al. (2017) also employed ultraviolet spectrophotometry to analyze the total flavonoids in four continuous cropping stubbles of 2-year-old Radix Hedysari, including normal, alternate, continuous, and three stubbles. The content of total flavonoids in Radix Hedysari from 2-year-old different crop stubbles was also examined. A comparable analysis revealed that, relative to that of normal stubble, the total flavonoid content of Radix Hedysari was reduced by 3.75% in alternate stubble, 20.00% in continuous stubble, and 36.25% in the three stubbles. These findings suggest that in the artificial cultivation of Radix Hedysari, gramineous crops serve as the optimal previous crop, whereas legumes are not as common as the previous crop. Bao et al. (2016) applied ultraviolet spectrophotometry to assess total flavonoids in 28 batches of Astragalus and Hedysari samples from various habitats in Gansu Province. Astragalus presented a greater average flavonoid content than Hedysari, with the highest flavonoid content observed in Hedysari from the Wudu area among the batches studied. This underscores the necessity of maintaining a clear distinction between the two, emphasizing that they should not be interchanged but rather employed independently for their respective purposes.

### 3.3 Colorimetric methods

The core principle underlying the colorimetric method is the interaction between flavonoids containing 3,5-phenolic hydroxyl groups, 4-carbonyl groups, or 3′, 4′-o-phenolic hydroxyl groups and metal ions, forming colored complexes. Aluminum salts, zirconium salts, and others are commonly employed as metal salts. Cheng et al. (2010) utilized the aluminum salt colorimetric method to determine the total flavonoid content in Radix Hedysari, demonstrating its applicability for such measurements. Ye et al. (2016) investigated six colorimetric methods (NaOH_2_-Al(NO_3_)_3_-NaOH, AlCl_3_, NaOH, phosphomolybdic acid, magnesium acetate methanol, and hydrochloric acid-magnesium powder) and ultraviolet spectrophotometry for evaluating the flavonoid content in Astragalus and Radix Hedysari polybotrys. These results revealed that while the colorimetric method was effective for quantifying flavonoids in Radix Hedysari polybotrys, ultraviolet spectrophotometry has emerged as the most appropriate method for accurately determining the total flavonoid content in both Radix Hedysari polybotrys and Astragalus.4 Chemical constituents.

**FIGURE 2:**
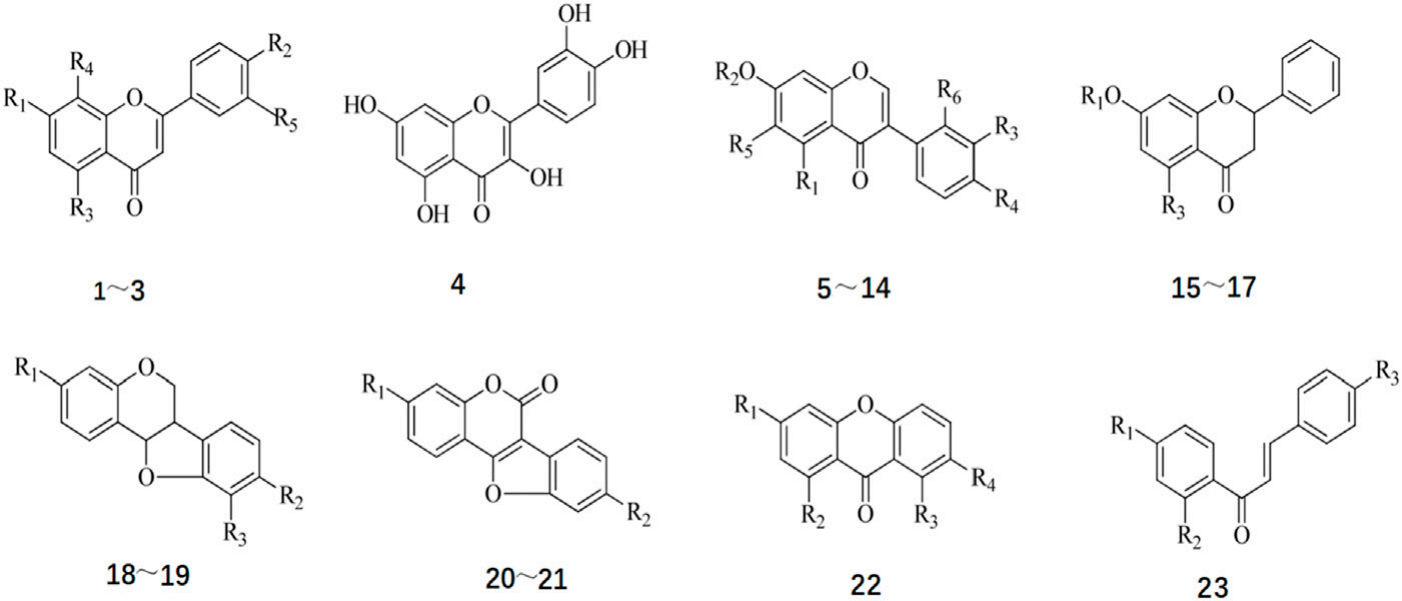
Chemical structure of Flavonoids in *Hedysari Radix* (Chinese Traditional and Herbal Drugs, 2021; 52(9).).

Flavonoids constitute essential active constituents of Radix Hedysari. Research has indicated that the core structure of flavonoids isolated from Radix Hedysari includes eight fundamental types (Liu, 2006): flavonoids, flavonols, isoflavones, dihydroflavones, pterocarpan, coumarin ether, ketone, and chalcone. The structural formula of Radix Hedysari flavonoids is shown in Figure 2, and the compound names and substituents are shown in Table 2.

**TABLE 2:** Flavonoids in *Radix Hedysari*.

Number	Compound name	Substituent group	Reference
1	7.4′-dihydroxyflavone	R_1_=R_2_=OH, R_3_=R_4_=R_5_=H	Zhao et al. (2015)
2	4′-methoxy-7-hydroxyflavone	R=OH.R_2_=OCH_3_, R_3_=R_4_=R_5_=H	Zhao et al. (2015)
3	Bongorientin	R_1_=R_2_=R_3_=Rs=OH, R_4_=Glc	Zhao et al. (2015)
4	Quercetin	R_1_=R_2_=R_3_=R_4_=OH	Zhao et al. (2015)
5	Calycosin	R_1_=R_3_=OH, R_2_=R_5_=R_6_=H. R_4_=OCH_3_	Zhao et al. (2015)
6	Calycosin-o-β-d-glucopyranoside	R_2_=Glu, R:=R;=OH, Rs=R_6_=H,R=OCH_3_	Zhao et al. (2015)
7	Onocol	R_1_=R_3_=R_5_=R_6_=H, R_2_=H, R_4_=OCH_3_	Zhao et al. (2015)
8	Formononetin-7-O-glucoside	R_1_=R_3_=R_5_=R_6_=H.R_2_=Glu.R_4_=OCH_3_	Zhao et al. (2015)
9	Genistein	R_1_=OH.R_2_=H, R_4_=OH, R_3_=R_5_=R_6_=H	Wang (2020)
10	Mangbinghuaxin-7-O-B-D - (6″- malonylmethyl) glucopyranose	R¡=Rз=Rs=R_6_=H, R_4_=OCH_3_, R_2_=COOHCHzCOOCH_2_-Glc	Liu et al. (2010)
11	Formononetin-7-0-β-D-glucose-6′ -malonate ethyl ester	R_1_=R_3_=R_5_=R_6_=H, R_4_=OCH_3_.R_2_=C_2_H_5_CO0CH_2_COOCH_2_-Glc	Liu et al. (2010)
12	3′, 7-dihydroxy-4′ -methoxyisoflavone	R_3_=OH.R_1_=R_2_=R_s_=R_6_=H, R_4_=OCH_3_	Toshio et al. (1984)
13	7-Hydroxy-4′.6-dimethoxyisoflavone	R_1_=R_2_=R_3_=R_6_=H.R_s_=R_4_=OCH_3_	Toshio et al. (1984)
14	vis voxel	R_1_=R_2_=R_3_=Rs=H.R_4_=OCH_3_.R_6_=OH	Toshio et al. (1984)
15	Liquiritigenin (7.4′-dihydroxyflavanone)	R_1_=H, R_2_=OH	Zhao et al. (2015)
16	Naringin	R_1_=Rut.R_2_=R_3_=OH	Zhao et al. (2015)
17	5,7,4′-Trihydroxyflavanone 5-O-β-D-glucopyranosyl-7-O-β-D-glucopyranoside	R_1_=Glu, R_2_=OH, R_3_=OGlu	Zhao et al. (2015)
18	3-hydroxy-9-methoxyrosesandalwood	R_1_=OH, R_2_=OCH_3_	Zhao et al. (2015)
19	3-hydroxy-9.10-dimethoxyroseane	R_1_=OH, R_2_=R_3_=OCH_3_	Wang (2020)
20	3-hydroxy-9-methoxycoumestan	R_1_=OH, R_2_=OCH_3_	Zhao et al. (2015)
21	3,9-Dimethoxycoumarin phenyl ether	R_1_=R_2_=OCH_3_	Zhao et al. (2015)
22	1,7-dihydroxy-3,8-dimethylxanthone	R_1_=R_2_=R_4_=OH.R_3_=OCH_3_	Zhao et al. (2015)
23	Isoliquiritigenin	R:=R2=R3=OH	Zhao et al. (2015)

## 4 Pharmacological effects

Research has demonstrated that Radix Hedysari flavonoids exhibit various pharmacological effects, including cytotoxic effects on tumor cells, anti-oxidation, anti-osteoporosis, fibrosis improvement, immunity enhancement, and a reduction in skeletal muscle damage. Consequently, in recent years, Radix Hedysari has been increasingly prescribed and widely utilized in clinical practice. The medicinal value of Radix Hedysari flavonoids has been confirmed, leading to their use in crafting diverse medicinal diets and their incorporation as medicinal and edible materials.

### 4.1 Cytotoxic mechanisms of Hedysari flavonoids in tumor cell models

Cancer is considered one of the most challenging diseases to manage in China. With the continuous development of science and technology, there is a growing range of treatment options for cancer. However, treatment often leads to significant adverse reactions in the human body, making cancer management difficult. Therefore, optimizing strategies for cancer prevention and treatment is particularly important. The isoflavones of Radix Hedysari constitute one of the main active ingredients of Radix Hedysari and are closely associated with its biological characteristics (Feng et al., 2021). Calycosin and formononetin are crucial index components for evaluating the quality of Radix Hedysari (Niu et al., 2021) and have therapeutic effects on liver cancer (Liu et al., 2021), gastric cancer (Wang and Zhao, 2021), non-small cell lung cancer (Yu et al., 2020), and cervical cancer (Wang et al., 2022a). Previous studies (Yu et al., 2020; Liu et al., 2021; Wang and Zhao, 2021; Wang et al., 2022a) have demonstrated Radix Hedysari flavonoids can be used as a potential therapeutic agent for gastric, leukemia, lung, and prostate cancer. The mechanism may involve inhibiting cell growth and proliferation, inducing apoptosis, and interfering with the cell cycle.

#### 4.1.1 Inhibition of cell growth and proliferation

Formononetin, an isoflavone constituent of Radix Hedysari polybotrys, dose-dependently inhibited prostate cancer cell proliferation *in vitro* at a concentration of 60 μmol/L. This inhibition was attributed to the induction of cell cycle arrest. Additionally, it markedly reduces the expression levels of cyclin-dependent 1 (cyclin D1) and cyclin-dependent kinase 4 (CDK4), leading to significant suppression of tumor growth in mice (Li et al., 2014b). Wang et al. (2007; 2012) investigated the impact of 80 μg/mL total flavonoids derived from Radix Hedysari on the proliferation of human chronic myelogenous leukemia K562 cells. These findings revealed a notable inhibitory effect of Radix Hedysari flavonoids on K562 cell growth. Furthermore, it upregulated the expression of the P21 gene while downregulating the expression of proliferating cell nuclear antigen (PCNA), thereby exerting cytotoxic effects on tumor cells.

#### 4.1.2 Induction of apoptosis

Calycosin, at a concentration of 50 μmol/L, has been demonstrated to decrease the expression of extracellular signal-regulated kinases, upstream nuclear factor-κB (NF-κB), and signal transducer and activator of transcription 3 (STAT3), in gastric cancer cells. This results in an increase in the expression of cytochrome C and B-cell lymphoma-2 (Bcl-2)-related cell death agonists, a decrease in the expression of the antiapoptotic protein Bcl-2, the induction of apoptosis (Zhang et al., 2021b). At a concentration of 25 μmol/L, formononetin, an isoflavone constituent of Radix Hedysari polybotrys, can activate the death receptor of the cysteine-aspartate protease (caspase) cascade. This activation mediates both exogenous and mitochondrial-dependent endogenous apoptotic pathways, leading to the apoptosis of pharyngeal squamous cell carcinoma cells and exerting cytotoxic effects on tumor cells. (Oh et al., 2020). Hu et al. (2019) reported that formononetin at a dose of 50 mg/kg could increase the number of positive human osteosarcoma U2OS cells expressing Bcl-2 associated X protein (Bax) and apoptotic protease activating factor-1 (Apaf-1). Moreover, it increased positive cells and the protein expression levels of Bax, Caspase-3, and Apaf-1 in U2OS tumor-bearing mice. Additionally, formononetin downregulates estrogen receptor alpha (ERα)- and phosphorylated protein kinase B (p-Akt)-positive cells and protein levels, facilitating apoptosis and demonstrating cytotoxic effects on tumor cells.

#### 4.1.3 Interference with the cell cycle

**FIGURE 3:**
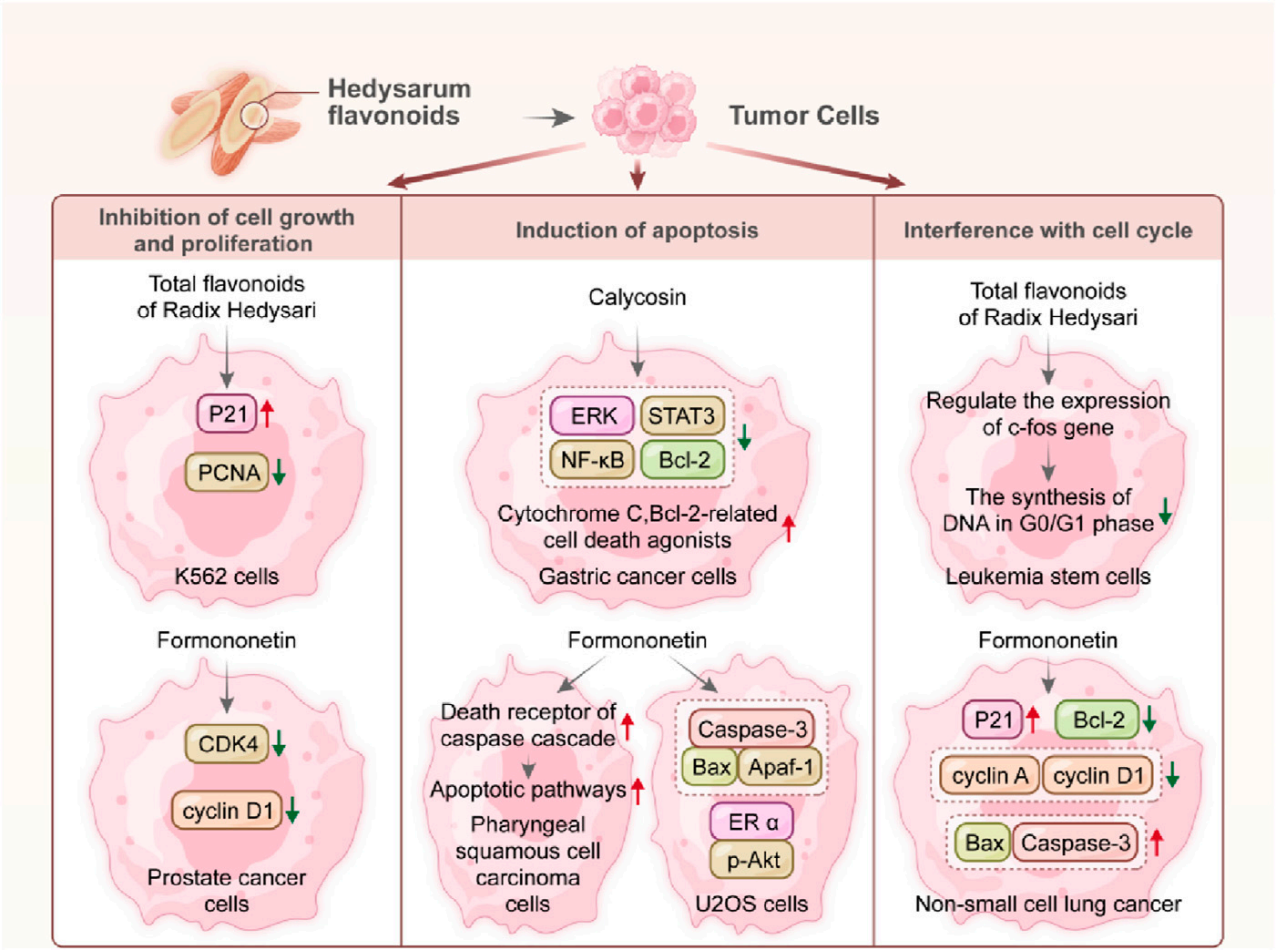
Cytotoxic effects on tumor cells of Radix Hedysari flavonoids.

Deng (2019b) investigated the impact of 80 μg/mL total flavonoids derived from Radix Hedysari on human leukemia. They discovered that these flavonoids hinder leukemia cell proliferation by regulating the expression of the c-fos gene and inhibiting DNA synthesis in the G0/G1 phase of leukemia stem cells. This mechanism contributes to the cytotoxic efficacy on leukemia stem cells of Radix Hedysari flavonoids, with the inhibitory effect showing a dose-dependent relationship. Isoflavones from Radix Hedysari polybotrys at a concentration of 150 μmol/L increase the expression of the p21 protein in non-small cell lung cancer cells (Deng, 2018). This reduces the expression of cell cycle regulatory proteins such as cyclin A and cyclin D1. Additionally, it (50, 100, 150 and 200 μmol/L) promotes the expression of Caspase-3 and the proapoptotic protein Bax while diminishing the expression of the antiapoptotic protein Bcl-2. Consequently, it induces cell cycle arrest and apoptosis in non-small cell lung cancer cells in the G1 phase, suggesting its potential as a preventive treatment for lung cancer (Yang et al., 2014). In conclusion, flavonoids from Radix Hedysari polybotrys primarily exert cytotoxic effects on tumor cells by inhibiting tumor cell growth, inducing tumor cell apoptosis, enhancing the body’s immune function, and synergizing with chemotherapeutic drugs, as illustrated in Table 3 and Figure 3.

**TABLE 3:** Cytotoxic effects on tumor cells of Radix Hedysari flavonoids.

Pharmacological action	Function	Model of study	Routes of administration	Drug dosage	References
Inhibit cell growth and proliferation	Reduced the expression levels of cyclin D1 and CDK4	Prostate cancer cell	Add the solution directly	60 μmol/L	Li et al. (2014b)
	Upregulated the expression of the P21 gene, downregulating the expression of PCNA.	Human chronic myelogenous leukemia K562 cells	Pre-dissolution method	80 μg/mL	Wang, 2007; Wang et al. (2012)
Induction of apoptosis	Decrease the expression of NF-κB and STAT3; upregulated the expression of protein Bcl-2	Gastric cancer cells	Pre-dissolution method	50 μmol/L	Zhang et al. (2021b)
	Activate the death receptor of the Caspase cascade	Pharyngeal squamous cell carcinoma cells	Pre-dissolution method	25 μmol/L	Oh et al. (2020)
	Enhance the number of positive cells expressing Bcl-2 associated X protein (Bax) and Apaf-1	Human osteosarcoma U2OS cells	Pre-dissolution method	50 mg/kg	Hu et al. (2019)
Interference with cell cycle	Regulating the expression of the c-fos gene and inhibiting DNA synthesis in the G0/G1	Leukemia stem cells	Add the solution directly	150 μmol/L 80 μg/mL	Deng (2018), 2019b
	Enhance the expression of the p21 protein; reduction in the expression cyclin A and cyclin D1; promotes the expression of Caspase-3 and Bax, diminishes the expression of Bcl-2	Lung cancer cells	Add the solution directly	50, 100, 150 and 200 μmol/L	Yang et al. (2014)

### 4.2 Antioxidation effects

Studies have demonstrated that flavonoids can scavenge free radicals and inhibit lipid peroxidation to achieve antioxidative pharmacological effects. These findings suggest flavonoids may be utilized as natural antioxidants in development and application. Chen et al. (2007) cultured human umbilical vein endothelial cells *in vitro* and applied H_2_O_2_ as a damaging agent to examine the protective effects of Radix Hedysari flavonoids against H_2_O_2_-induced endothelial cell damage. Compared with those in the normal group, the lactate dehydrogenase (LDH) levels in the culture medium of the cells were significantly elevated after exposure to H_2_O_2_ (100 μmol/L) for 4 h. The flavonoids from Radix Hedysari decreased the release of LDH in the cells induced by H_2_O_2_, indicating a protective role against cellular injury. Additionally, each dose of Radix Hedysari flavonoids increased superoxide dismutase(SOD) activity and markedly reduced malondialdehyde (MDA) levels in the cells, suggesting potential antiperoxidative damage properties. Xue (2018) reported that both Radix Hedysari and Radix Astragali seu Hedysari decoctions exhibited strong antioxidant activity *in vitro* and improved D-galactose-induced oxidative stress in a dose-dependent manner. Zhao et al. (2022) reported that a 95% ethanol extract of Radix Hedysari presented the highest antioxidant activity compared with other extraction solvents, with significant quantities of formononetin, pterocarpin, and formononetin monomers, demonstrating a dose-effect relationship between the formononetin content and antioxidant activity. Yang et al. (2019) optimized the flavonoid extraction process from Radix Hedysari, further confirming its robust antioxidant capability *in vitro*. Yuan et al. (2019b) optimized the ultrasonic extraction process of total flavonoids from Radix Hedysari via an orthogonal method. Antioxidant activity was used as an index, and a 1–10 mg/mL solution of total flavonoids from Radix Hedysari exhibited promising antioxidant capacity *in vitro*, demonstrating a clear dose-effect relationship. Furthermore, Hedysari flavonoids also displayed significant antioxidant activity *in vivo*. The total flavonoids of Radix Hedysari polybotrys were found to have a protective effect on H_2_O_2_-induced umbilical vein endothelial cell injury. Each dose significantly inhibited malondialdehyde injury, reduced LDH release and the intracellular malondialdehyde content, and increased LDH and superoxide dismutase (SOD) activity. The underlying mechanism may involve scavenging oxygen free radicals and enhancing the antioxidant capacity of umbilical vein endothelial cells (Chen et al., 2007). Wang et al. (2020) demonstrated that 5 μmol/L orientin could activate nuclear factor E2 related factor 2 (Nrf2), thereby improving the antioxidant effect by promoting the expression and translocation of Nrf2, resulting in reduced expression of heme oxygenase-1 (HO-1) in cells, maintaining a low level of oxidative stress, and thus achieving an antioxidant effect. In summary, Hedysari flavonoids exhibit significant antioxidant activity both *in vitro*, and this activity may be attributed to the regulation of LDH, SOD, malondialdehyde, and other enzymes, as well as the expression of antioxidant-related factors. Additionally, the discovery of the antioxidant activity of flavonoids from Radix Hedysari may provide new insights for developing healthcare and beauty products.

### 4.3 Improvement in pulmonary fibrosis

Pulmonary fibrosis is an extremely complex and challenging respiratory disease characterized by fibroblast proliferation, significant accumulation of the extracellular matrix, and destruction of the lung tissue structure. Currently, the drugs commonly used to mitigate pulmonary fibrosis in clinical settings are primarily hormones (Meyer, 2017), which are associated with numerous adverse reactions (Raghu et al., 2011) and high costs (Huang and Xu, 2022). Research has indicated that traditional Chinese medicine has distinct advantages in preventing and treating pulmonary fibrosis. Both individual and combined traditional Chinese medicines can ameliorate pulmonary fibrosis through targeted regulation of related signaling pathways (Li et al., 2024). Numerous studies have shown that Radix Hedysari flavonoids significantly enhance pulmonary fibrosis treatment and perform better than Astragalus flavonoids do.

Zhang et al. (2014) assessed the effects of total flavonoids from Radix Hedysari on transforming growth factor-β1 (TGF-β1) protein expression and lung tissue ultrastructure in rat models of pulmonary interstitial fibrosis induced by bleomycin at a dosage of 5 mg/kg. They reported that doses of 7.5, 15.0, and 22.5 mg/kg Radix Hedysari flavonoids could suppress TGF-β1 expression, significantly alleviate pathological damage, and reduce extracellular matrix deposition, with the highest dose showing the most pronounced effects. Li et al. (2013) reported the impact of total flavonoids from Radix Hedysari polybotrys on pulmonary function in similar rat models. These results indicated improvements in pulmonary function, dynamic compliance, the volume index, and the volume flow index at doses of 7.5, 15.0, and 30.0 mg/kg, suggesting a potential antifibrotic effect of Radix Hedysari polybotrys, although further investigation is needed to elucidate the underlying mechanisms involved. Lin et al. (2020) explored the influence of Radix Hedysari administered at 37.41 mg/kg via aerosols (3.5–4.0 mg/m^3^ for 40 min) on pulmonary fibrosis. Notably, Radix Hedysari improved pulmonary fibrosis, significantly reducing hyaluronic acid and laminin (LN) levels in lung tissue, with aerosol administration showing enhanced effectiveness. The comparative effects of 15 mg/kg Astragalus and Hedysari flavonoids on lung function in rat models of pulmonary interstitial fibrosis were also examined, revealing that both inhibited the disease process, with Hedysari flavonoids demonstrating superior efficacy (Lin et al., 2013). Su et al. (2016) demonstrated that Radix Hedysari flavonoids at 7.5, 15.0, and 22.5 mg/kg could mitigate pulmonary fibrosis by reducing alveolar inflammation and inhibiting collagen fiber proliferation and deposition, thus decreasing hyaluronic acid and LN levels in lung tissue. The therapeutic effects of these flavonoids were greater than those of polysaccharides and saponins from Radix Hedysari. Wang et al. (2022c) reported that 7.5, 15.0, and 30.0 mg/kg Radix Hedysari polybotrys flavonoids could counteract bleomycin-induced pulmonary interstitial fibrosis in rats by reducing hyaluronic acid, LN, and hydroxyproline (HYP) levels in lung tissue, thereby improving this condition (She et al., 2014). She Li et al. (2014a) reported that Radix Hedysari flavonoids at 22.5, 15.0, and 7.5 mg/kg improved pulmonary fibrosis in a dose-dependent manner.

**FIGURE 4:**
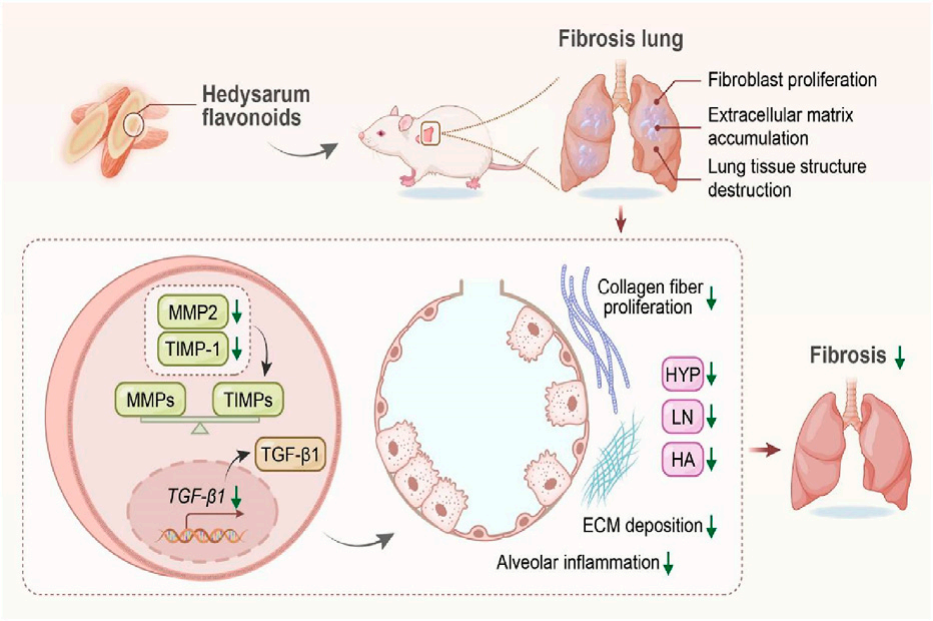
Mechanism by which Radix Hedysari flavonoids improve pulmonary fibrosis.

Su et al. (2015) established a model of pulmonary fibrosis through intratracheal instillation of bleomycin and the administration of Radix Hedysari flavonoids at 22.5, 15.0, and 7.5 mg/kg over 28 days, with samples collected at 7, 14, and 28 days for analysis. These findings showed that Radix Hedysari polybotrys flavonoids could inhibit the expression of matrix metalloproteinase 2 (MMP2) and tissue inhibitor of metalloproteinase-1 (TIMP-1), contributing to a balance between MMPs and TIMPs and suppressing the progression of pulmonary fibrosis. In summary, the mechanism by which Radix Hedysari flavonoids inhibit pulmonary fibrosis involves a reduction in extracellular matrix deposition and the inhibition of collagen fiber proliferation, among other processes, as depicted in Figure 4.

### 4.4 Anti-osteoporosis

The prevalence of osteoporosis in China is increasing due to population aging and improved living standards. This condition, characterized by decreased bone mass and degradation of the bone microstructure, is attributed primarily to greater osteoclast activity than osteoblasts, resulting in greater bone resorption (Jia et al., 2022). The declining physiological structure and various life indicators of elderly individuals increase their susceptibility to diseases, significantly impacting their quality of life (Dong et al., 2018). Despite the numerous adverse drug reactions encountered in clinical osteoporosis treatment (Chen et al., 2018a), traditional Chinese medicine is promising because of its minimal adverse effects and proven efficacy (Wang et al., 2023). Plant-derived active compounds such as polysaccharides and flavonoids have emerged as potential solutions for treating osteoporosis (Zhao and Li, 2019). These active substances regulate bone-specific matrix proteins, transcription factors, signaling pathways, and other targets to treat osteoporosis through various mechanisms while minimizing adverse reactions (Azam et al., 2020). They have garnered significant attention and are generally well accepted by patients, as evidenced by Guo et al. after analyzing the etiology and pathogenesis of osteoporosis in traditional Chinese medicine (Chi et al., 2023). Hedysari flavonoids have significant preventive and therapeutic effects on osteoporosis induced by various factors.

#### 4.4.1 Promotion of the proliferation and differentiation of osteoblasts

Fang (2020) examined the influences of Radix Hedysari polysaccharides and flavonoids on the osteogenic differentiation of rat bone marrow stromal cells (rBMSCs) and rat calvarial osteoblasts (ROBs). These findings revealed that calycosin, a component of Radix Hedysari isoflavones, contributed to activating the insulin-like growth factor-1 receptor (IGF-1R)/phosphoinositide 3-kinase (PI3K)/Akt signaling pathway. Specifically, 1 × 10^−6^ mol/L calycosin significantly enhanced the osteogenic differentiation of rBMSCs and ROBs, outperforming Radix Hedysari polysaccharide. Additionally, the effects of five flavonoids—calycosin, formononetin, isoliquiritigenin, and pterocarpin (1 × 10^−9^, 1 × 10^−8^, 1 × 10^−7^, 1 × 10^−6^, and 1 × 10^−5^ mol/L)—on the cell activity and osteogenic-related factors of rBMSCs and ROBs were investigated (Fang et al., 2019a). Notably, these flavonoids increased the proliferation of rBMSCs and ROBs, increased alkaline phosphatase (ALP) activity, increased Ca^2+^ levels, and increased the area and number of calcified nodules, contributing to anti-osteoporosis effects. Chen et al. (2018b), in their research on the spectrum-effect relationship of the anti-osteoporosis effects of active components in *Radix Hedysari*, reported that 10 μmol/L of calycosin, an isoflavone, enhanced ALP activity in osteoblasts and promoted osteoblast differentiation, thereby providing anti-osteoporosis benefits. Wu (2010) reported that calycosin exhibited significant bone protection in ovariectomized rats by analyzing its preventive and therapeutic effects at 15 and 30 mg/kg doses. Quercetin, a plant flavonoid in Radix Hedysari known for its estrogenic effects, was studied by Pang et al. (2018), who treated BMSCs with 2.5 μmol/L quercetin. Quercetin can increase ALP activity, promote the production and mineralization of the extracellular matrix, and increase the expression of Runt-related transcription factor 2 (RUNX2), an osteoblast-specific transcription factor, and osteopontin. Furthermore, quercetin regulates the expression of osteoblast-specific genes through estrogen receptors and activates the bone morphogenetic protein (BMP)/Smad signaling pathway (Li et al., 2019a). These findings suggest that quercetin promotes the differentiation of BMSCs into osteoblasts via an estrogen receptor-mediated pathway, with the BMP/Smad pathway playing a crucial role. Zhang et al. (2020) reported that quercetin at 5 μmol/L promoted the proliferation and osteogenic differentiation of BMSCs by inhibiting the microRNA-206 (miR-206) pathway and increasing the expression of connexin 43.

#### 4.4.2 Reduction in bone loss

**FIGURE 5:**
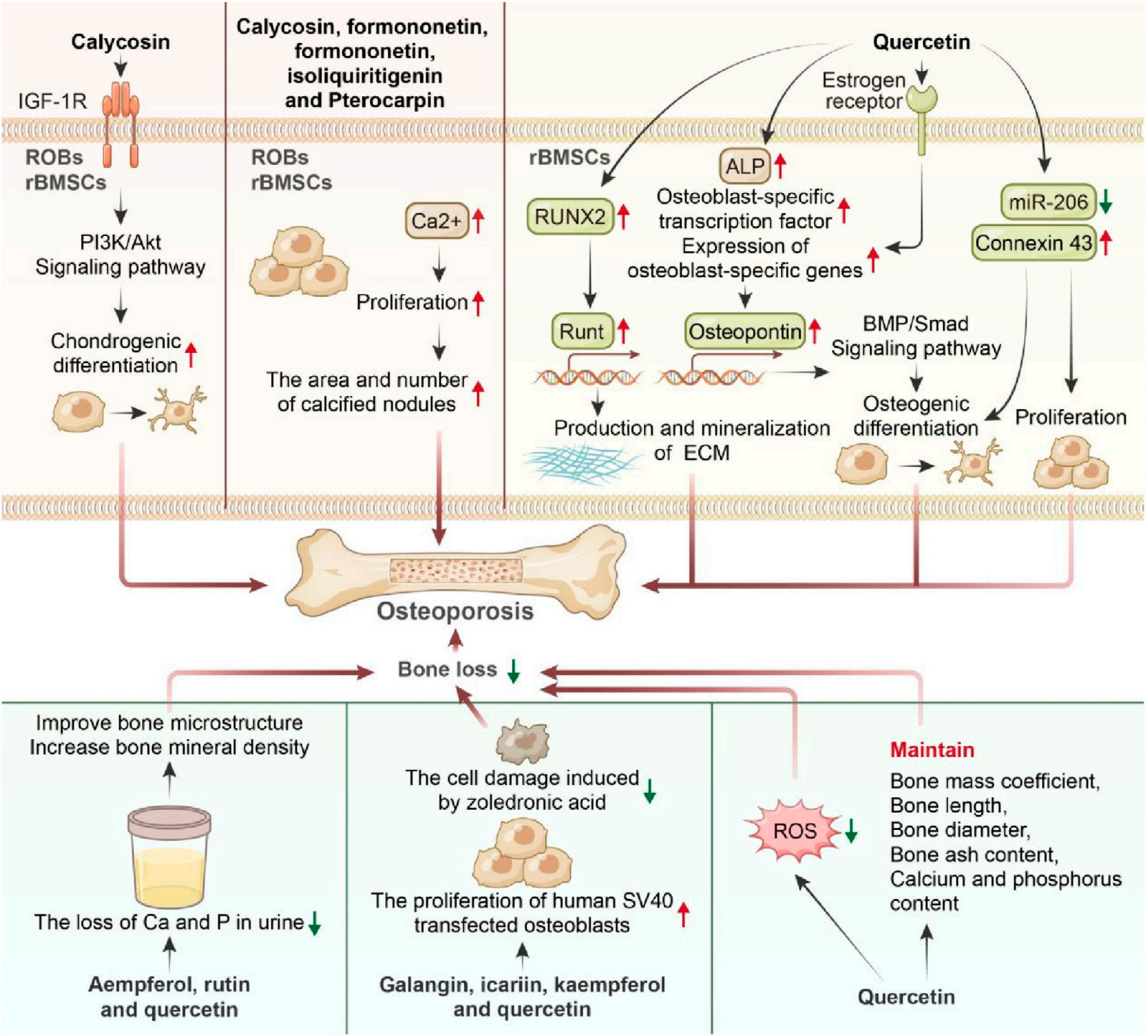
Anti-osteoporosis mechanism of Hedysari flavonoids.

Yuan et al. (2018) reported that administering kaempferol, rutin, and quercetin were administ-ered by gavage to rats at a dosage of 50 mg/kg resulted in decreased excretion of Ca and P in urine, along with improved bone microstructure and increased bone mineral density. Notably, kaempferol had the most significant effect. Kim et al. (2018) demonstrated that flavonoids such as galangin, icariin, kaempferol, and quercetin (10, 25, 50, 100 μmol/L) could increase the proliferation of human SV40-transfected osteoblasts. Additionally, these compounds mitigated the cell damage induced by zoledronic acid, with galangin and kaempferol showing notable cytoprotective effects against zoledronic acid. Another study revealed that quercetin at a dosage of 100 mg/kg improved the bone mass coefficient, bone length, bone diameter, bone ash content, and calcium and phosphorus contents reduced by retinoic acid while reducing retinoic acid-induced oxidative stress and bone loss (Oršolić et al., 2014). It can be inferred that the calycosin in Radix Hedysari may stimulate bone cell differentiation, contributing to its pharmacological anti-osteoporosis effects. In summary, the mechanism by which Radix Hedysari flavonoids improve osteoporosis likely involves promoting osteoblast proliferation and differentiation, reducing calcium loss, increasing bone density, maintaining bone equilibrium, and enhancing bone quality for osteoporosis prevention and treatment (Table 4; Figure 5).

**TABLE 4:** Anti-osteoporosis mechanism of Hedysari flavonoids.

Pharmacological action	Function	Model of study	Routes of administration	Drug dosage	References
Promote the proliferation and differentiation of osteoblasts	Activating the IGF-1R/PI3K/Akt signaling pathway; enhanced ALP activity, raised Ca2+ levels, and expanded the area and number of calcified nodules	rBMSCs and ROBs	Pre-dissolution method	1 × 10^−9^, 1 × 10^−8^, 1 × 10^−7^, 1 × 10^−6^, and 1 × 10^−5^ mol/L	Fang et al., 2019a; Fang (2020)
	Increases ALP activity in osteoblasts and fosters osteoblast differentiation	Osteoblasts	Pre-dissolution method	10 μmol/L	Chen et al. (2018b)
	Boost ALP activity, encourage the production and mineralization of the extracellular matrix, and increase the expression of RUNX2; activated the BMP/Smad signaling pathway	BMSCs	Pre-dissolution method	2.5 μmol/L	Pang et al. (2018), Li et al. (2019a)
	Inhibiting the miR-206 pathway and enhancing the expression of connexin 43	BMSCs	Pre-dissolution method	5 μmol/L	Zhang et al. (2020)
Reduce bone loss	Decreased excretion of Ca and P in urine, along with improved bone microstructure and increased bone mineral density	SD rats	Intragastric administration	50 mg/kg	Yuan et al. (2018)
	Enhance the proliferation of human SV40-transfected osteoblasts	Osteoblasts	Pre-dissolution method	10, 25, 50,100 μmol/L	Kim et al. (2018)
	Improved bone mass coefficient, bone length, bone diameter, bone ash content, calcium, and phosphorus content; reducing retinoic acid-induced oxidative stress and bone loss	Osteoblasts	Pre-dissolution method	100 mg/kg	Oršolić et al. (2014)

### 4.5 Alleviation of skeletal muscle injury

Skeletal muscle plays a critical role in human posture and movement, yet injuries to this tissue are common (Kwee and Mooney, 2017). Currently, nonsteroidal anti-inflammatory drugs are commonly prescribed to alleviate pain and inflammation post-muscle injury and restore muscle function (Bu and Wang, 2022), although treatment options remain somewhat limited (Jones et al., 2015). Jones et al. (2015) demonstrated that, unlike paracetamol, nonsteroidal anti-inflammatory drugs (NSAIDs) have no effect on pain at 1–2 h or at 2–3 days and may have no effect on pain at seven or more days. NSAIDs may result in a small increase in gastrointestinal adverse events and may make no difference in neurological adverse events compared with paracetamol. Yuan et al. (Yuan, 2023) demonstrated that administering 40 mg/kg orientin effectively reduced skeletal muscle inflammation and oxidative stress in rats with exercise-induced skeletal muscle injury. Notably, the level of p38 mitogen-activated protein kinase (MAPK) phosphorylation in the skeletal muscle of the experimental group was lower than that in the control group. Furthermore, orientin facilitated the transcription and translocation of Nrf2 in rat skeletal muscle with exercise-induced injury by inhibiting the transcription and expression of Kelch-like ECH-associated protein 1 (Keap1), likely involving the p38 MAPK pathway and the Nrf2/Keap1 pathway. Compared with muscle injury, quercetin/β-cyclodextrin gel treatment reduces lipid peroxidation and SOD and catalase activity, thereby diminishing skeletal muscle inflammation and oxidative stress and consequently mitigating muscle injury (Sousa Filho et al., 2021). Administering total flavonoids of Radix Hedysari at a dose of 0.2 mg/kg delayed the accumulation of reactive oxygen species, malondialdehyde, and LDH in rat skeletal muscle following eccentric exercise while increasing SOD activity, indicating significant preventive and therapeutic effects on skeletal muscle injury induced by oxidative stress after high-intensity exercise (Ma et al., 2015). Duan et al. (2016) noted that administering total flavonoids of Radix Hedysari at a dose of 0.2 mg/kg moderately slowed the decline in nitric oxide content in rat skeletal muscle after eccentric exercise, suggesting its potential to scavenge oxygen free radicals, reduce malondialdehyde and LDH content, and increase SOD activity, thus mitigating skeletal muscle injury. Yang et al. (2021) demonstrated that centrifugal exhaustive exercise activated NF-κB signaling molecules in rat skeletal muscle cells, initiating apoptosis. Administering total flavonoids from Radix Hedysari (0.2 mg/kg) alleviated the stress response induced by high-intensity exercise, delaying skeletal muscle fatigue and injury after eccentric exercise. This effect may involve reducing NF-κB, Bcl-2/Bax, cytochrome C, and caspase-3 expression levels in skeletal muscle tissue and inhibiting skeletal muscle cell apoptosis. Ma et al. (2015) reported that administering total flavonoids of Radix Hedysari delayed the accumulation of reactive oxygen species (ROS), MDA, and LDH in rat skeletal muscle following eccentric exercise while increasing SOD activity, indicating its potential to prevent skeletal muscle damage caused by oxidative stress after high-intensity exercise. In summary, these findings suggest that the total flavonoids of Radix Hedysari may alleviate or prevent skeletal muscle injury by scavenging oxygen free radicals; reducing ROS, MDA, and LDH contents; increasing SOD activity; and reducing the expression levels of NF-κB, Bcl-2/Bax, Cyt c, and Caspase-3 in skeletal muscle tissue.

### 4.6 Anti-atherosclerosis effects

Atherosclerosis, a prevalent cause of coronary heart disease, ischemic cerebrovascular disease, and other conditions, poses a significant threat to human health and life expectancy, with its incidence steadily increasing in recent years (Liu et al., 2022). Epidemiological investigations have revealed that flavonoids can exert antiatherosclerotic effects by reducing oxidation (Wang et al., 2018), preventing thrombosis, enhancing endothelial function, and regulating blood lipid levels (Mahmoud et al., 2019).

Chen et al. (2008) conducted experiments in which they cultured human umbilical vein endothelial cells *in vitro* and induced damage via oxidized low-density lipoprotein (ox-LDL) to investigate how total flavonoids of Radix Hedysari could counteract ox-LDL-induced endothelial cell injury. These results indicated that these flavonoids (20, 40, 60, 80, 100 mg/L) reduced the release of LDH in umbilical vein endothelial cells, suggesting a protective effect against ox-LDL-induced damage. Additionally, they reported that flavonoids increased nitric oxide synthase (NOS) activity in ox-LDL-damaged endothelial cells, leading to increased synthesis and secretion of nitric oxide (NO), suggesting a potential antiatherosclerotic role through the modulation of NO levels. Flavonoids are known to exert anti-inflammatory effects by modulating the NF-κB pathway, either through inhibitory factor κB kinase regulation or through interference with NF-κB binding to DNA (Mackenzie et al., 2004). For example, quercetin at 100 μmol/L inhibits NF-κB activation through p38 kinase inhibition, thereby reducing atherosclerosis in mice (Cho et al., 2003). Research has also shown that flavonoids such as quercetin at 2 mmol/L and rutin can block the platelet membrane glycoprotein GPIIb/IIIa receptor, preventing platelet activation and calcium ion carrier aggregation, indicating antiplatelet activity (Zaragozá Arnáez et al., 2021). Recent studies suggest that quercetin at 1.5 mmol/L can inhibit cyclooxygenase by up to 90%, primarily by altering arachidonic acid metabolism to interfere with platelet aggregation (Zaragozá Arnáez et al., 2022). An evaluation of the antiatherosclerotic effects of citrus flavonoids in different cell models revealed that 75 μmol/L naringenin inhibited cholesterol ester transfer protein and microsomal triglyceride transfer protein in human hepatoma cells (Borradaile et al., 2003), thereby limiting cholesterol ester and triglyceride availability in lipoprotein formation (Borradaile et al., 2002). In summary, the mechanism underlying the antiatherosclerotic effects of Radix Hedysari flavonoids is shown in Table 5.

**TABLE 5:** Anti-atherosclerosis mechanism of Radix Hedysari flavonoids.

Pharmacological action	Function	Model of study	Routes of administration	Drug dosage	References
anti-oxidation	The activity of NOS in umbilical vein endothelial cells was increased, and the intracellular NO was increased	human umbilical vein endothelial cell	Add the solution directly	20, 40, 60, 80, 100 mg/L	Chen et al. (2008)
anti-inflammatory	Regulating NF-κB pathway	T cells and macrophage	Pre-dissolution method	100 μmol/L 1, 0.5, 0.1, and 0.05 mmol/L	Cho et al. (2003), Mackenzie et al. (2004)
Prevent thrombosis	Blockade of platelet membrane glycoprotein GPIIb/IIIa receptor	volunteer blood	Calcium ionophore (CI A23187)	2 mmol/L	Zaragozá Arnáez et al. (2021)
Interfere with platelet aggregation	Change arachidonic acid	—	—	1.5 mmol/L	Zaragozá Arnáez et al. (2022)
blood lipid regulation	Inhibition of cholesteryl ester transfer protein and microsomal triglyceride transfer protein	HCC Cell	Add the solution directly	75 μmol/L	Borradaile et al. (2002), Borradaile et al. (2003)

### 4.7 Anti-hepatic fibrosis

Liver fibrosis represents a reparative response to damage, where liver injury from diverse causes can trigger its onset. Processes such as hepatocyte necrosis, apoptosis, inflammatory cell infiltration, and alterations in the extracellular matrix induced by liver injury promote the development of liver fibrosis. If left untreated, liver fibrosis can progress to cirrhosis and even liver cancer, significantly impacting human health (Jia et al., 2023a). Given that liver fibrosis is reversible in its early stages while cirrhosis is irreversible, inhibiting or reversing liver fibrosis represents a crucial approach in chronic liver disease treatment. Consequently, the prevention and treatment of liver fibrosis are key areas of research both domestically and internationally. Flavonoids, abundant in various plants, are found in numerous traditional Chinese medicines used clinically for treating liver fibrosis (Ma et al., 2022).

Zhang et al. (2021a) revealed that administering 80 mg/kg calycosin effectively suppressed carbon tetrachloride-induced liver fibrosis in mice. This inhibition is likely due to the activation of the JAK2/STAT3 pathway by calycosin, which enhances the protein expression of Janus kinase 2 (JAK2) and STAT3. Deng et al. (2018) reported that 200 μmol/L calycosin inhibited the proliferation, activation, and migration of liver fibrosis cells induced by TGF-β1, possibly by binding to calycosin and subsequently reducing the level of intracellular estrogen receptor β5 (ERβ5). Moreover, quercetin at concentrations ranging from 8 to 128 μmol/L significantly suppressed hepatic stellate cell proliferation over 0–72 h, promoting apoptosis, likely through modulation of the Wnt/β-catenin signaling pathway to counter hepatic fibrosis (Feng, 2017). Quercetin at 15 mg/kg was found to inhibit the Wnt/β-catenin pathway by decreasing β-catenin and Wnt protein expression, thus preventing liver fibrosis progression and providing liver protection (Chen et al., 2021). Furthermore, 50 mg/kg quercetin affects the Notch1 pathway by reducing the expression of the neurogenic gene Notch homologous protein 1 (Notch1), leading to the inhibition of M1 polarization and the induction of anti-inflammatory and anti-hepatic fibrosis effects (Li et al., 2018). Naringin at doses of 15 and 30 mg/kg suppressed collagen proliferation and alleviated hepatic fibrosis by reducing alanine aminotransferase (ALT), aspartate amino transferase (AST), and HYP contents, significantly mitigating the increase in the carbon tetrachloride-induced liver coefficient and inhibiting collagen proliferation and hepatic fibrosis (Wang and Qian, 2014). Hedysari flavonoids inhibit liver fibrosis by suppressing the proliferation, activation, and metastasis of liver fibrosis cells, along with collagen fiber proliferation. Additionally, Chen et al. (2015) highlighted the pharmacological effects of Radix Hedysari in preventing and treating liver fibrosis, with adenosine and calycosin exhibiting potent efficacy. However, many active ingredients in Hedysari remain unexplored and necessitate further investigation. These studies collectively underscore that Hedysari flavonoids can prevent liver fibrosis by impeding the proliferation, activation, and metastasis of liver fibrosis cells, along with collagen fiber proliferation.

### 4.8 Immunomodulatory effects

Radix Hedysari polybotrys flavonoids exert immunomodulatory effects mainly by enhancing the expression of immune cells and immune organs and activating immune-related pathways. Hedysari flavonoids can regulate the body’s immune function and enhance the body’s nonspecific immune and specific immune response. Studies have shown that various flavonoids can promote the phagocytosis of macrophages, increase the activity of natural killer (NK) cells, and regulate the proliferation and differentiation of T cells and B cells and that the regulation of various immune indicators is comprehensive and significant.

#### 4.8.1 Increased expression of immune cells

Flavonoids play an immunomodulatory role mainly by enhancing lymphocyte function, increasing lymphocyte number, and promoting stem cell differentiation. Related studies have shown that (Si et al., 2021; Chen et al., 2022) flavonoids can promote the proliferation of mouse spleen lymphocytes, increase the proliferation of T and B lymphocytes and the secretion of cytokines, and improve the immune response of mice. Flavonoids can also inhibit the mitosis of immune cells, inhibit the secretion of immunoglobulins (IgM, IgA, and IgG) by B lymphocytes, promote the proliferation of spleen lymphocytes, and promote the secretion of interleukin(IL)-2, thereby increasing the killing effect of NK cells. The results revealed that flavonoids have a two-way immunoregulatory effect. Wang et al. (2022b) reported that in a model of bleomycin-induced pulmonary fibrosis in mice, various flavonoids can improve alveolar inflammation, inhibit TGF-β1, reduce collagen fiber deposition in the extracellular matrix, reduce lesion range, and improve lung tissue function by increasing the proportion of T lymphocyte subsets, which is comparable to the effects of prednisone in the clinic. Mesenchymal stem cells (MSCs) are pluripotent stem T cells with immunomodulatory properties and strong immunomodulatory effects (Hazrati et al., 2022; Tang et al., 2022). Radix Hedysari flavonoids can promote the differentiation of rat bone marrow mesenchymal stem cells (BMSCs), promote the proliferation of calcified nodules of osteoblasts, significantly increase the degree of cell mineralization, increase the bone strength of mice (Fang et al., 2019b), and improve the body’s immunity. These findings suggest that Hedysari flavonoids can be developed into excellent osteogen cell-inducing factors and applied to the clinical treatment of metabolic bone diseases.

#### 4.8.2 Promotion of the differentiation of central immune organs

The total flavonoids of Radix Hedysari affect the differentiation of central immune organs. It can promote the proliferation and differentiation of human promyelocytic leukemia cells (HL-60), regulate the expression of proto-oncogenes (C-fos), control the number of cells in the gap phase, reduce DNA synthesis and transcription, induce cell differentiation, inhibit the growth of cancer cells (Deng, 2019a), and have a certain therapeutic effect on leukemia.

#### 4.8.3 Regulation of inflammatory factors and immune regulation-related pathways

The immunomodulatory effects of flavonoids depend on anti-inflammatory and related pathways. For example, total flavonoids can significantly inhibit the synthesis of IL-6 and reduce the expression of the proinflammatory factors IL-1β and tumor necrosis factor(TNF)-α. It can also reduce the protein and mRNA levels of the TLR4 and NF-κB pathways in the cartilage tissue of collagen-induced arthritis mice, inhibit the TLR4/NF-κB signaling pathway, reduce the inflammatory response, and treat autoimmune diseases (Li et al., 2021; Shen et al., 2021; Xie and Li, 2022). By downregulating the level of ROS, activating the protein kinase C (PKC) pathway, regulating the mitogen-activated protein kinase (MAPK) signaling pathway, regulating autophagy, inducing the apoptosis of cancer cells, inhibiting the biological activity of cancer cell proliferation, and regulating the expression of pathway-related proteins to exert immunomodulatory effects (Reyes-Farias and Carrasco-Pozo, 2019; Wang et al., 2019; Xu et al., 2019; Wei et al., 2024), there are certain tumor immune effects.

### 4.9 Other pharmacological effects

In addition to their known pharmacological effects, such as anti-oxidation effects, improvements in pulmonary fibrosis, anti-tumor effects, anti-osteoporosis effects, and reductions in skeletal muscle injury, the flavonoids found in Radix Hedysari polybotrys also exhibit anti-inflammatory, hypoglycemic, and antirenal fibrosis properties. Yao et al. (2022) reported that formononetin at a 50 mg/kg dosage significantly decreased the serum urea nitrogen, serum creatinine, and blood glucose levels in diabetic nephropathy (DN) rats. Moreover, varying degrees of improvement were observed in inflammation and fibrosis in the glomeruli, suggesting a substantial enhancement in renal function among diabetic rats treated with formononetin. The mechanism may involve the activation of the adenosine monophosphate-activated protein kinase (AMPK)/silent information regulator 1 (SIRT1)/forkhead box protein O1 (FoxO1) pathway to promote DN autophagy and reduce kidney damage. Additionally, formononetin significantly reduced abnormal glucose metabolism and renal damage in DN rats and inhibited renal fibrosis. This effect may be attributed to the activation of AMPK and SIRT1 expression by formononetin, the suppression of FoxO1 expression, the amelioration of renal oxidative stress, and the inhibition of autophagy, consequently alleviating the onset and progression of DN.

## 5 Conclusion and prospects

In summary, hedysariflavone is the primary active compound in the traditional Chinese medicine Radix Hedysari and is known for its multiple components and targets. It exhibits a broad spectrum of pharmacological activities and is essential for treating contemporary illnesses. This study investigated seven key pharmacological effects of Radix Hedysari flavonoids: cytotoxic effects on tumor cells, anti-oxidation, anti-osteoporosis, pulmonary fibrosis improvement, skeletal muscle injury reduction, antiatherosclerosis, and antihepatic fibrosis effects. As a recognized medicinal material in Gansu Province, Radix Hedysari offers high quality, substantial yields, and distinctive harvesting and processing techniques. Although limited, recent studies on Radix Hedysari flavonoids suggest that they outperform the clinically used Radix Astragali in anti-oxidation and pulmonary improvement, thus offering significant potential for development and clinical use. Investigations into the extraction and separation of flavonoids from Radix Hedysari, along with component identification and pharmacological activity assessment of the extracts, have confirmed the extensive applications and developmental prospects of Radix Hedysari, positioning it as a Chinese herbal medicine with substantial potential for growth. Furthermore, flavonoids, which are secondary plant metabolites, are prevalent in many traditional Chinese medicines and exhibit diverse pharmacological properties. Radix Hedysari contains various flavonoids, including isoflavones, calycosin, formononetin, the flavonol quercetin, and the dihydroflavone naringin, which contribute to its anti-oxidation, cytotoxic effects on tumor cells, and pulmonary improvement effects. Notably, calycosin and formononetin are crucial for assessing the quality of Radix Hedysari and have notable effects on liver cancer, gastric cancer, non-small cell lung cancer, and cervical cancer. Therefore, more systematic, detailed, and comprehensive studies on the extraction, separation, content determination, and pharmacology of Radix Hedysari flavonoids are imperative for future research.

Additionally, the current research has several shortcomings: 1) the clinical application of Radix Hedysari, whether as a single agent or a compound, remains limited; 2) studies on Radix Hedysari flavonoids are confined to the cellular and animal levels, with the specific pharmacodynamic foundation of these flavonoids not adequately defined; 3) reports on the structural modification of Radix Hedysari flavonoids are scarce; and 4)*In vitro* experiments cannot serve as direct evidence for disease treatment. Consequently, future research should extend the scope and depth of studies on Radix Hedysari flavonoids, investigate whether various Radix Hedysari flavonoids modifiers can enhance their immunomodulatory functions, and further examine the pharmacological effects of active Radix Hedysari ingredients derived from traditional prescriptions. It is also critical to explore the biological activity resulting from more structural modifications of Radix Hedysari polybotrys flavonoids and to compare the effectiveness of different administration methods. Clarification of the mechanisms underlying its cytotoxic effects on tumor cells, fibrosis improvement, and atherosclerosis inhibition is needed. Additionally, while Hedysari flavonoids display various pharmacological effects, precise dosage control requires further investigation. The types and concentrations of chemical components in Chinese medicinal materials are crucial quality indicators and guide clinical medication choices (Liu et al., 2023). Research on the clinical applications of *Radix Hedysari* in traditional Chinese medicine, as well as its integration with Western medical practices, continues to be a major focus of both current and future investigations. Thus, investigations of the pharmacological effects and clinical applications of the flavonoid monomer components of Radix Hedysari are vital to provide a theoretical basis for its large-scale cultivation and clinical use.

Although Hedysari flavonoids have shown significant efficacy in alleviating various diseases at the cellular and animal levels, existing studies have focused mainly on animal models or cell experiments and have not yet entered clinical trials or markets. The reasons for this phenomenon may include the following: ① Insufficient safety and toxicological data exist. Although Hedysari flavonoids have shown significant efficacy at the cellular and animal levels, safety and toxicological data for humans are lacking, which is an indispensable part of the drug development process. ② Clinical trial design and regulatory challenges: Traditional drugs may face special challenges in clinical trial design, including determining appropriate doses, evaluating long-term efficacy and safety, and meeting regulatory requirements. ③ Quality control issues: the extraction and purification process of Radix Hedysari flavonoids may be difficult to standardize, resulting in inconsistent product quality, which affects the reliability and repeatability of clinical trial results. ④ Capital and resource constraints: Clinical trials usually require considerable money and resources, whereas traditional drugs may have difficulty attracting sufficient investment, especially in the early stages. ⑤ Intellectual property and patent issues: Intellectual property protection of traditional drugs may be more complex, which may affect enthusiasm for drug development and commercialization processes. ⑥ Culture and market acceptance: traditional medicines may need to overcome cultural differences and market acceptance, especially when promoted globally.

Meanwhile, this manuscript elaborates on the three major pathways of the cytotoxic effects of Hedysari flavonoids in tumor cell models, namely, inhibiting tumor proliferation, inducing apoptosis, and arresting the cell cycle. It also explains that these cytotoxic effects are associated with mechanisms such as reducing the expression levels of cyclin D1 and CDK4, decreasing the expression of NF-κB and STAT3, upregulating the expression of protein Bcl-2, regulating the expression of the c-fos gene, and inhibiting DNA synthesis in the G0/G1 phase (see Table 3; Figure 3). However, it must be clearly recognized that inhibiting tumor proliferation, inducing apoptosis, or arresting the cell cycle *in vitro* is not equivalent to the mechanism of anti-tumor action. “Anti-tumor” faces more complex clinical issues (such as inhibiting metastasis, prolonging survival, etc.), and the two cannot be equated. There are a series of problems with *in vitro* experiments, such as false positives caused by non-specific cytotoxicity induced by high concentrations, differences in drug concentrations between *in vitro* and *in vivo* experiments, differences in the timing of action between *in vitro* experiments and *in vivo* dynamics, the absence of the tumor microenvironment, and the lack of pharmacokinetics. These factors mean that Hedysari flavonoids cannot yet be considered as anti-tumor agents. In the future, multidimensional preclinical validation will be needed to establish their effectiveness in tumor treatment. However, the cytotoxic effects of Hedysari flavonoids on tumor cells show potential and promising anti-tumor capabilities, which give us reason to expect that they may be a potential anti-tumor drug.

In addition, the correlation between the traditional efficacy of Radix Hedysari and the pharmacological effects of flavonoids has attracted considerable attention. As a traditional Chinese medicine, Radix Hedysari has a long history of medicinal use. It has always been regarded as a good product for nourishing qi and blood, tonifying the kidney and essence, invigorating the spleen, appetizing, consolidating the surface and stopping sweating. Modern pharmaceutical research has further revealed the pharmacological basis of Radix Hedysari, especially the significant role of its flavonoids in cytotoxic effects on tumor cells, anti-oxidation, improvement of fibrosis and other aspects. The following will discuss in detail the correlation between the traditional efficacy of Radix Hedysari and the pharmacological effects of its flavonoids.

Radix Hedysari, the dry root of the leguminous plant Radix Hedysari polybotrys, is traditionally used to treat symptoms such as qi depression, superficial deficiency and spontaneous sweating, qi deficiency edema, and qi deficiency and blood failure. Its ability to tonify qi and nourish blood is particularly prominent and can be used to improve symptoms such as anemia, fatigue, and shortness of breath. In the theory of traditional Chinese medicine, Qi is the driving force of life activities, and the generation and operation of blood depend on the promotion of Qi. Radix Hedysari polybotrys can increase the vitality of the body and improve the body’s disease resistance by nourishing qi and blood (Zhang et al., 2013).

As one of the main active components, hedysari flavonoids have significant antioxidant effects. Free radical peroxidation has direct or indirect effects on human health. Hedysari flavonoids can inhibit lipid peroxidation, effectively scavenge free radicals, and protect cells from oxidative damage. This mechanism not only helps delay aging but also provides a theoretical basis for the treatment of oxidative damage-related diseases. The antioxidant activity of flavonoids from Radix Hedysari polybotrys has been confirmed in several studies. For example, Zhao et al. (2022) reported that flavonoids from a 95% ethanol extract of Radix Hedysari polybotrys had significant antioxidant activity. The DPPH and ABTS· + free radical scavenging rates were 91.07% and 97.70%, respectively, and the iron ion reduction ability was 0.416. This antioxidant effect is complementary to the traditional efficacy of Radix Hedysari in tonifying qi and nourishing blood and jointly maintains the health status of the body. Free radical peroxidation has direct or indirect effects on human health. Radix Hedysari polybotrys flavonoids can inhibit lipid peroxidation, effectively remove free radicals, and protect cells from oxidative damage. This is consistent with the theory that traditional Chinese medicines tonify qi to increase the body’s vitality and delay aging.

Hedysari flavonoids, owing to their pronounced and effective cytotoxic effects on tumor cells, could potentially serve as therapeutic agents for the prevention and treatment of malignancies including gastric cancer, leukemia, lung cancer, and prostate cancer. Its mechanism may involve inhibiting cell growth and proliferation, inducing apoptosis and interfering with the cell cycle. The use of this component may provide new ideas and methods for cancer treatment (Jia et al., 2023b). In traditional Chinese medicine, the formation of tumors is often related to qi deficiency, blood stasis, phlegm block and other factors. Radix Hedysari can improve the internal environment of the body by tonifying qi and nourishing blood, promoting blood circulation and removing blood stasis, thus inhibiting the growth and development of tumors. Therefore, the cytotoxic effects on tumor cells of Radix Hedysari flavonoids is closely related to their traditional effects of tonifying qi and nourishing blood, promoting blood circulation and removing blood stasis.

In terms of improving fibrosis, Hedysari flavonoids also have significant pharmacological effects. Fibrotic diseases such as pulmonary fibrosis, myocardial fibrosis, and renal fibrosis are extremely complex and refractory. Their pathological features include fibroblast proliferation, accumulation of a large amount of extracellular matrix, and destruction of tissue structure. Hedysari flavonoids can reduce extracellular matrix deposition and inhibit collagen fiber proliferation, thereby significantly improving the degree of pathological damage caused by fibrotic diseases (Zhang et al., 2024). This mechanism of action is related to the traditional efficacy of Radix Hedysari, which includes water swelling, solid surfaces, and antiperspiration. In traditional Chinese medicine, symptoms such as edema and spontaneous sweating are often related to factors such as qi deficiency and water-dampness retention. Radix Hedysari polybotrys can regulate the body’s water metabolism and improve the pathological state of fibrotic diseases by tonifying qi and consolidating the surface, promoting diuresis and detumescence.

In addition, Radix Hedysari flavonoids also have pharmacological effects, such as anti-osteoporosis, reducing skeletal muscle damage, anti-atherosclerosis, immune regulation, and anti-inflammatory and hypoglycemic effects (Fu et al., 2024). These mechanisms are closely related to the traditional efficacy of Radix Hedysari. For example, Radix Hedysari can strengthen muscles and bones and prevent osteoporosis by tonifying the kidney, replenishing qi and nourishing blood; by invigorating the spleen and promoting digestion, it can increase the body’s nutritional absorption capacity, thereby reducing skeletal muscle damage; by promoting blood circulation to prevent blood stasis and regulating blood lipids, atherosclerosis can be prevented; by replenishing qi and nourishing blood, regulating immunity, it can increase the body’s resistance to disease, thus exerting anti-inflammatory effects; and by regulating glucose metabolism, it can assist in lowering blood sugar and improving the symptoms of diabetic patients.

In summary, there is a close relationship between the traditional efficacy of Radix Hedysari and the pharmacological effects of its flavonoids. The pharmacological effects of flavonoids from Radix Hedysari, such as cytotoxic effects on tumor cells, anti-oxidation, fibrosis improvement, anti-osteoporosis, skeletal muscle injury reduction, antiatherosclerosis, immune regulation, anti-inflammatory and hypoglycemic effects, not only provide a new direction for modern pharmaceutical research but also provide a scientific basis for the traditional application of Radix Hedysari. Through in-depth studies of the pharmacological mechanism of flavonoids in Radix Hedysari, we can better understand and use Radix Hedysari, a traditional Chinese herbal medicine, and make greater contributions to human health.

In the future, with the continuous development of science and technology, our understanding of flavonoids in Radix Hedysari will be more in depth. Through modern scientific and technological means, we can further reveal the mechanism of action of flavonoids in Radix Hedysari, optimize their extraction process, improve their bioavailability, and develop safer, more effective and convenient pharmaceutical preparations. Moreover, we can combine the flavonoids of Radix Hedysari polybotrys with other traditional Chinese medicine components to form a synergistic traditional Chinese medicine compound, which provides more options for the treatment of various diseases. In the near future, the flavonoids of Radix Hedysari may play a more important role in the field of medicine.

To meet these challenges, first, strengthening basic research, especially in pharmacology and toxicology, is necessary to provide a sufficient scientific basis for the entry of Hedysari flavonoids into the clinic and market. Second, work closely with regulatory agencies to ensure that the clinical trial design meets the requirements and that the necessary approval is obtained. Third, the extraction and purification process should be optimized to ensure the consistency and standardization of product quality. Fourth, financial support from the government, academic institutions, and the private sector should be sought to overcome financial constraints. Fifth, the protection of intellectual property rights should be strengthened, and the innovation and development of traditional drugs should be reasonably rewarded. Sixth, market research should be conducted, the needs of different cultures and markets should be understood, and effective marketing strategies should be formulated. Through the implementation of these measures, the possibility of Radix Hedysari polybotrys flavonoids entering clinical trials and markets can be improved, and ultimately, more treatment options for patients worldwide can be provided.
